# Electrical wire as a foreign body in a male urethra: a case report

**DOI:** 10.1186/1752-1947-3-49

**Published:** 2009-02-03

**Authors:** Konstantinos G Stravodimos, Georgios Koritsiadis, Georgios Koutalellis

**Affiliations:** 1 1st Department of Urology, University of Athens Medical School, Laiko Hospital, Athens, Greece

## Abstract

**Introduction:**

Self-inflicted foreign bodies in the male urethra and urinary bladder are an emergency that urologists may rarely have to face. A case of an electrical wire inserted in the male urethra and coiled in the bladder is presented.

**Case presentation:**

A 53-year-old male presented with the inability to void and bloody urethral discharge after having introduced an electrical wire in his urethra for masturbation 3 hours earlier. He had made several unsuccessful attempts to remove it.

**Conclusion:**

The variety of these objects may be impressive and removal of the foreign body may be quite challenging requiring imagination and high-level surgical skills., In this case an electrical wire was used and the diagnostic as well as the therapeutic steps for its removal are presented.

## Introduction

Self-insertion of foreign bodies into the male urethra and urinary bladder for autoerotic stimulation is a rather rare emergency condition that an urologist may encounter. A case of an electrical wire inserted in the male urethra and coiled in the bladder is presented.

## Case presentation

A 53 year old male presented with the inability to void and bloody urethral discharge after having introduced an electrical wire into his urethra for masturbation 3 hours earlier. He had made several unsuccessful attempts to remove it.

**Figure 1:**
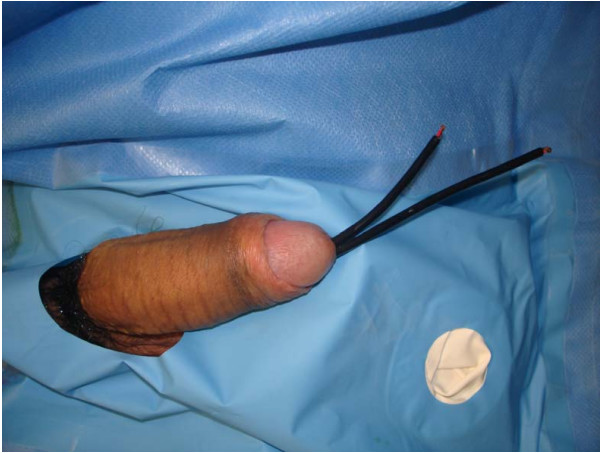
**Two ends of the wire outside the urethral meatus**.

**Figure 2:**
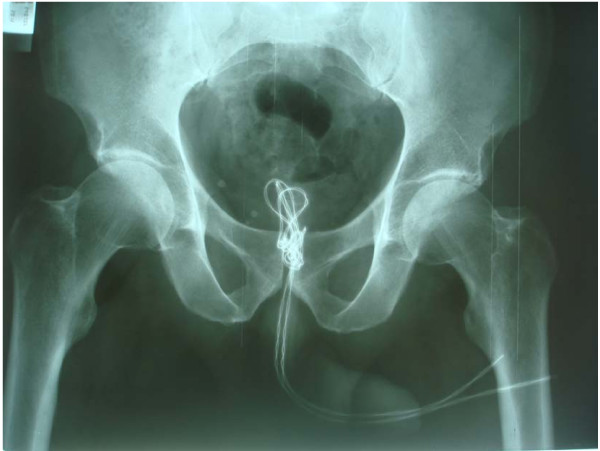
**X-ray of kidney, ureter, bladder: coiled up radiopaque wire inside the bladder**.

**Figure 3:**
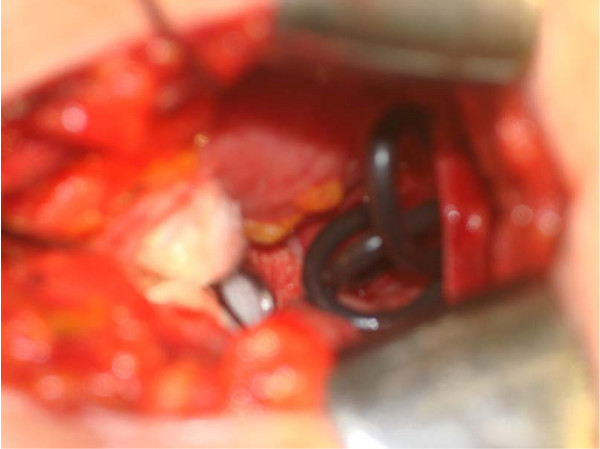
**Intra-operative view through a suprapubic cystotomy**.

During the physical examination, the two ends of the wire were observed in the urethral meatus (Figure 1). An x-ray of kidney, ureter, bladder (KUB) demonstrated a coiled up radiopaque wire inside the bladder (Figure 2). The patient was married with children and his wife accompanied him. His socioeconomic status was of upper class. It was the first time he had ever self-inflicted a foreign body in his urethra and he had no history of psychiatric illness or drug addiction. After giving his formal consent, the patient was taken to the operating room. Under general anesthesia and fluoroscopic control, an unsuccessful trial was made to pull the wire. An attempt was made to insert a 22Fr cystoscope or an 8Fr ureteroscope parallel to the wire but this was impossible due to lack of space. Then a suprapubic cystotomy was performed and the wire was removed (Figure 3). The patient was discharged on the third postoperative day and the urethral catheter was removed on the sixth day. He was on intravenous antibiotics for three days and on a per os regimen for another week. On the six month evaluation, the patient is well with a normal uroflow and no symptoms of urethral stricture.

## Discussion

A large number of self-inflicted foreign bodies have been reported in the male urethra and urinary bladder [1-5]. The variety of these objects is really impressive, including sharp and lacerating objects (e.g needle, pencil, wire), wire-like objects (cable, rubber tube), parts of animals (bones) or plants and vegetables (hay, cucumber), fluids (e.g, glue) and powders (e.g, cocaine) [1].

The most common reason for self-insertion of a foreign body into the male urethra is of erotic or sexual nature, especially masturbation or sexual gratification [1-4]. A mental illness or drug intoxication may also be the reason [1,2]. Masturbation in males is very frequent with a rate close to 100% [6]. In the majority of cases, the patient feels guilty and humiliated [1,2], therefore he postpones the search for medical help. In our case, the patient was expressing repentance for his action. A few very interesting psychiatric-psychoanalytic theories have been postulated. According to Kenney's theory, the initiating event is the coincidentally discovered pleasurable stimulation of the urethra, followed by repetition of this action with objects of unknown danger, driven by a particular psychological predisposition to sexual gratification [1,7]. Wise considered urethral manipulation as a paraphilia combining sadomasochistic and fetishistic elements where the orgasm of the individual depends on the presence of the fetish. He believed it shows a regression to a urethral stage of erotism due to a traumatic event or a strong libidinal drive [1,8]. From the clinical view, many authors advocate the psychiatric evaluation of these patients, based on theories that consider this act as an indication of an impulsive behavior, self-punishing in nature that may aggravate to suicide [1]. The psychiatric evaluation is controversial as many of these patients are psychologically normal [2]. In our case, as there is no psychiatrist in our hospital, a neurological evaluation was performed revealing no signs of depression or impulsive behavior.

Clinical presentation may vary from asymptomatic to swelling of external genitalia, dysuria, poor urinary stream or retention, bloody or purulent urethral discharge and ascending urinary tract infection [1,2].

Depending on the type of foreign body and its location, various methods of removal have been described, including meatotomy, cystoscopy, internal or external urethrotomy, suprapubic cystostomy, Fogarty catheterization, and injection of solvents. Removal of the foreign body may be quite challenging requiring imagination and high-level surgical skills. Endoscopic therapy is the standard. The most suitable method is relevant to the size and mobility of the object. In the majority of mobile objects inside the urethra, the mobility is towards the bladder where, after having been pushed, the foreign body can be grasped by forceps or retrieval baskets. Nephroscopes have been used for the retrieval of screws as well as magnetic retrievers for galvanic objects [1]. The YAG laser has also been used lately [5]. In cases where endoscopic procedures are unsuccessful, then open surgery is recommended. For objects stuck in the penile urethra, external urethrotomy is recommended [9], while for intravesical foreign bodies, a suprapubic cystotomy is the treatment of choice.

## Conclusion

A self-inflicted foreign body in the urethra and bladder is a rare situation. Endoscopic manipulation is the preferred first-line treatment and if unsuccessful, open procedures may be necessary.

## Competing interests

The authors declare that they have no competing interests.

## Authors' contributions

KS was the patient's surgeon and has been involved in drafting the manuscript and revising it critically for important intellectual content. GKoritsiadis has made contributions to conception and design. GKoutalellis contributed to the analysis and interpretation of data and was also the doctor who examined the patient in the emergency room. All authors read and approved the final manuscript.

## Consent

Written informed consent was obtained from the patient for publication of this case report and accompanying images. A copy of the written consent is available for review by the Editor-in-Chief of this journal.
